# Effects of patient education on the quality of life of patients with type 2 diabetes mellitus: A scoping review

**DOI:** 10.51866/rv.208

**Published:** 2022-11-25

**Authors:** Amirah Mustapa, Maria Justine, Haidzir Manaf

**Affiliations:** 1 PhD, Integrative Pharmacogenomics Institute, Universiti Teknologi MARA, Puncak Alam Campus, Puncak Alam, Selangor, Malaysia. Email: haidzir5894@uitm.edu.my; 2 MSc, Department of Physical Rehabilitation Sciences, Kulliyyah of Allied Health Sciences, International Islamic University Malaysia, Kuantan Campus, Kuantan, Pahang, Malaysia.; 3 PhD, Centre for Physiotherapy Studies, Faculty of Health Sciences, Universiti Teknologi MARA, Puncak Alam Campus, Puncak Alam, Selangor, Malaysia.

**Keywords:** Health education, Health promotion, Patient education, Type 2 diabetes mellitus, Quality of life

## Abstract

**Introduction:**

Patient education is an integral component of diabetes mellitus care. The emergence of different methods and characteristics of patient education has led to varying outcomes of quality of life (QoL). Herein, we systematically searched for published studies reporting patient education and its methods and characteristics for improving the QoL of patients with type 2 diabetes mellitus (T2DM).

**Method:**

In this scoping review, eligible studies from six databases (PubMed, Scopus, Cochrane Library, Springer Link, Science Direct and Google Scholar) were identified. The keywords used in the search strategies were as follows: health education, health promotion, patient education, diabetes care, QoL, diabetes mellitus and type 2 diabetes mellitus. Two reviewers independently screened all references and full-text articles retrieved to identify articles eligible for inclusion.

**Results:**

A total of 203 articles were identified in the initial search. Of them, 166 were excluded after screening the titles and abstracts. Further full-text screening led to the subsequent removal of 22 articles, leaving 15 articles eligible for data extraction.

**Conclusion:**

There is a broad array of methods of patient education for improving the QoL of patients with T2DM. Self-management education with supplementary supervision and monitoring effectively improves QoL. Future studies must emphasise the application of holistic education covering psychological distress, diet plan, and physical health.

## Introduction

Currently, the worldwide prevalence of diabetes mellitus (DM) is increasing tremendously regardless of age and disease type. About 463 million people, representing 9.3% of the global adult population aged between 20 and 79 years in 2019, have DM.^1,2^ This figure is predicted to increase extensively to about 578 million (10.2%) in 2030 and 700.2 million (10.9%) in 2045.^1,2^ This alarming prevalence of DM substantially impacts medical costs and has emerged as a global health burden.

DM is a disproportionately expensive disease, with an estimated annual global expenditure of USD 760 billion for diabetes care in 2019.^3^ The yearly expenditure is projected to increase to approximately USD 825 billion by 2030 and USD 845 billion by 2045, which is primarily driven by the increasing prevalence of type 2 diabetes mellitus (T2DM).^3,4^ T2DM accounts for 90 to 95% of all cases of DM.^1^ Therefore, the present review focused on T2DM, as it is more common than type 1 DM.

Patients with DM generally experience a poor quality of life (QoL) owing to DM-related complications such as foot ulceration, amputation, and retinopathy.^5,6^ Thorough and explicit patient education on T2DM can avert and delay the progression of the disease. Evolving epidemiological studies have also elucidated the effectiveness of patient education on lifestyle modification, well-balanced diet, weight loss, smoking cessation and regular exercise in controlling glucose homoeostasis and improving QoL.^7-10^ Patient education is also an integral component of DM care.^10^

Patient education on T2DM is a supportive and self-motivating process that involves imparting knowledge, abilities and skills in DM self-care, increasing self-efficacy and motivation, changing lifestyle and behaviour, improving compliance to therapeutic regimens, improving awareness of DM-related complications and enhancing emotional resilience, ultimately improving QoL.^10-12^ Current educational practices have shifted from the didactic style concentrating on the attainment of knowledge to interventions involving patient participation.^13^

The outcomes of patient educational techniques are influenced by their mode, method and delivery, which eventually impact QoL.^13,14^ Failure of educational techniques for T2DM decreases the perceived susceptibility to the complications of DM.^15,16^ Moreover, the emergence of different methods and characteristics of patient education has led to varying outcomes of QoL. In this review, we systematically searched for published studies reporting patient education and its methods and characteristics for improving the QoL of patients with T2DM.

## Methods

The review protocol has been registered within the Open Science Framework (https://osf. io/3gk6v). This scoping review was conducted using the methodological framework proposed by Arksey and O’Malley.^17^ The article identification and screening processes were synthesised using the Preferred Reporting Items for Systematic Reviews and MetaAnalyses Extension for Scoping Reviews (PRISMA-ScR).^18^ Electronic-based searches were performed in six databases (PubMed, Scopus, Cochrane Library, Springer Link, Science Direct and Google Scholar) to identify existing studies that described the methods and characteristics of patient education for improving the QoL of patients with T2DM.

The comprehensive search of articles (except for systematic and narrative reviews) published from 2010 to 2022 was conducted between 2 and 6 February 2021. Studies were excluded when they were not reported in the English language. The keywords used in the search strategies were as follows: health education, health promotion, patient education, diabetes care, quality of life, diabetes mellitus and type 2 diabetes mellitus. The Boolean terms ‘AND’, ‘OR’ and ‘AND NOT’ were used to separate the keywords.

Thereafter, two reviewers independently screened all references and full-text articles retrieved to identify articles eligible for inclusion. Discrepancies between the reviewers were resolved via consensus. Finally, two reviewers independently extracted data from the selected articles and input them in tabulation form in a structured Excel sheet. The following data were extracted: name of author(s), year of publication, country, method, characteristics of the intervention (period, mode, delivery, content, contact hour and provider), outcome measures of QoL and their significance and adverse events throughout the intervention. Two other reviewers further checked the Excel sheet to identify any errors in data extraction. The data were summarised on the basis of the method of the included study, characteristics of patient education, outcome measures of QoL, adverse events and adherence to patient education.

## Results

**Figure 1:**
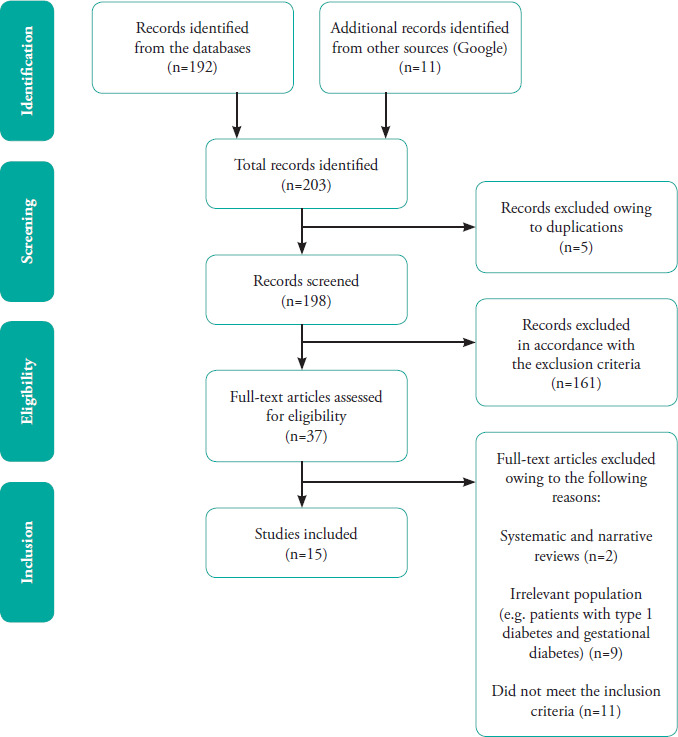
PRISMA-ScR flowchart of the scoping review.

A PRISMA-ScR flow diagram showing the stages of article screening and selection is shown in **Figure 1**. A total of 203 articles were identified from the databases and other sources, including Google Scholar. Of them, 166 were excluded after screening the titles and abstracts. Further full-text screening led to the subsequent removal of 22 articles, leaving 15 articles eligible for data extraction.^19-32^

### Study description

**Table 1** shows the characteristics of the included studies. In total, 2,909 patients with T2DM were allocated into an intervention group and a control group. The sample sizes ranged from 10 to 298 and from 10 to 210 in the intervention and control groups, respectively, except for the studies by Baraz et al.,^11^ Mahmoud et al.^31^ and Wong et al.^24^ that did not utilise a control group. Two studies included patients with T2DM on insulin and/ or oral hypoglycaemic therapy.^20,31^ Meanwhile, Estuningsih et al.^27^ grouped their hospitalised participants according to nutritional education before the intervention.

Five studies designated both intervention and control groups to receive usual DM care.^19-22,27^ In the study by Estuningsih et al.,^27^ all participants received nutritional consultation before the intervention. In the study by Pon et al.,^21^ all participants had access to an online care platform (e-Vita) to support their self-management skills. In the study by Sherifali et al.,^22^ all participants received usual DM education along with community resources and an accelerometer.

Four studies used Diabetes Self-Management Education and Support (DSME/S),^19,20,29,30^ while three studies used distinct group-based patientcentred and/or self-management education programmes such as Next Education (NEED),^26^ the Chronic Disease Self-Management Program (CDSMP),^28^ and the Patient Empowerment Programme (PEP).^24^

### Intervention characteristics

The intervention duration ranged from 4 weeks to 4 years, except in the study by Pon et al.^21^ For the intervention group, three studies did not report the mode of educational programme as either individual or group.^11,25,27^ Three studies used individual-based educational programmes^19,20,22^; nine, group-based educational programmes^19,21,23,24,26,29-32^; one, a classroom-based educational programme^28^; and twelve, face-to-face educational programmes.^19,21,23-32^ The other studies used educational technology,^11^ text messages via a smartphone application^20^ and phone calls^22^ for the delivery of the educational programmes.

Regarding intervention content, 10 studies focused on self-management or self-care skills.^11,19-22,24,25,28-30^ Christoffersen et al.^26^ tailored their educational programme based on the participants’ needs, focusing on psychosocial issues and behavioural changes and aiming to achieve a balanced life. Meanwhile, Estuningsih et al.^27^ applied intensive monitoring using a self-regulated learning approach with four sessions of educational meeting that comprised basic information about DM and a diet programme, obstacles encountered during the diet programme and implementation of the diet programme and assessed the effectiveness of the intervention. Mahmoud et al.^31^ conducted a psychoeducational programme using various interactive educational methods, including counselling, demonstration, group discussion and vignette.

Mostafa et al.^32^ utilised an educational programme in the Arabic language, including discussion and feedback using lectures (knowledge) and videos (practical) that consisted of definitions, causes, types, signs and symptoms, treatments, complications and nursing managements of DM. Trento et al.^23^ assigned group care in three phases: (1) introduction, (2) main content regarding role-playing with real-life simulations expressing patients’ opinions and life experiences with DM and (3) summary. Two studies enhanced the content regarding self-efficacy in their educational programmes.^19,24^

The contact hour for the intervention ranged from 55 minutes to 3.5 hours per session, which was held either weekly or monthly or at 3-to-4-month intervals, except in the study by Boels et al.^20^ in which each participant received one text message per day two to six times per week. For the educational providers, all included studies employed highly trained healthcare professionals such as dietitians, nurses, physicians, DM specialists and endocrinology specialists, except for the studies by Estuningsih et al.^27^ and Jahromi et al.^29^ in which educators and coordinators conducted the educational programmes without specifically mentioning whether they had been trained.

### Outcome measures

The most commonly used outcome measurement tools for QoL were the World Health Organization Quality of Life Scale,^19,27,29,32^ Diabetes-Dependent Quality of Life Questionnaire,^20,22^ Diabetes Quality of Life/ Mod Scale^23^ and EuroQoL Five-Dimensional Scale (EQ-5D-3L/5L).^20,21,25,26^ Other studies used the Iranian Short-Form Health Survey,^11^ Self-Reported Health-Related Quality of Life Scale,^28^ Diabetes Quality of Life Brief Clinical Inventory,^30^ Short-Form 12 and 36 Health Surveys Version^2,20,24^ Short-Form SixDimensional Scale^24^ and RAND 36-Item Health Survey 1.0.31 Estuningsih et al.^27^ further measured QoL objectively based on the blood glucose level, cholesterol level, blood pressure and body mass index.

### Adverse events and compliance

Two studies reported no serious adverse events or withdrawals during the intervention.^19,20^ Azami et al.^19^ further reported that none of their participants were hospitalised or died due to hypoglycaemic events. Meanwhile, a study reported that 60 participants visited the emergency department or were hospitalised during the >1-year monitoring period but found no significant difference between the intervention and control groups.^22^ Additionally, six studies reported moderate-to-good compliance, satisfaction and response of the participants to the interventions.^19,22,24,25,30,31^

In contrast, Boels et al.^20^ observed poor compliance towards the use of written diaries in recording the glucose level. Pon et al.^21^ further noted a low participation rate among their patients with T2DM.

### QoL

Eight studies found significant differences in the QoL in the intervention group depending on the domains measured.^22,24,25,27,29-32^ Conversely, seven studies found either a nonsignificant slight or great improvement in the QoL in the intervention group.^11,19-21,23,26,28^

**Table 1:** Characteristics of the included studies.

Author	Country	Patient	Method	Intervention						Outcome measure of QoL	Significance of the outcome measure of QoL
				Period	Mode	Delivery	Content	Contact	Provider		
Azami et al.^19^	Iran	IG: 71 CG: 71	- Usual DM care (e.g. self-care management, lifestyle modification and medication adherence) - Intervention for 20 to 30 minutes per session at 3-month intervals - Pamphlets **IG:** Plus DSME - Booklets - Movie clips and group discussions - Weekly follow-up phone call (2 months after the group discussion)	12 weeks	Group	F2F	Booklet: Diet, PA, medication, blood glucose level monitoring, foot care and healthy living with DM Movie clips: Information about T2DM, prevention ofcomplications, PA, daily foot care, healthy eating and healthy living with DM Group discussion: Building knowledge, SE and skills regarding self-goal setting, action planning, problem-solving, sharing and peer support	Movie clips: 10 minutes weekly on the 1st month Group discussion: 2 hours weekly on the 1st month	DSME: Multidisciplinary Group discussion: DM specialist nurse	WHOQO L-BREF Baseline 3 months 6 months	Effect of time × group: ↑↓ Between-group and time: ↔ Non-significant trend towards greater improvements in QoL
Baraz et al.^11^	Iran	30	Self-care educational programme	12 weeks	Not stated	Educational technology	Self-care educational programme: Advice on diet, medicine, skills, glycaemic control, PA and nutrition	55 minutes for 2 sessions	Endocrinology specialists	Iranian Short-Form Health Survey Baseline 3 months	General health: ↑↓ Physical function: ↑↓ Physical role: ↑↓ Emotional role: ↔ Social performance: ↑↓ Physical pain: ↑↓ Vital strength and energy: ↔ General health perception: ↔ Overall QoL: ↔
Boels et al.^20^	Netherlands	Patients with T2DM on insulin therapy IG: 115 CG: 115	Usual care IG: Plus DSME/S CG: Plus a smartphone application after completing follow-up	24 weeks	Individual	Text messages via a smartphone application	DSME/S: Hypoglycaemia, dietary habits, PA and glucose control	One text message per day 2 to 6	Text messages critically reviewed by a dietician, physiotherapist, practice nurse and two patients with T2DM on insulin therapy	DDQoL EQ-5D-5L SF-36 Baseline 6 months 9 months	DDQoL: ↔ EQ-5D-5L: ↔ SF-36: ↔ Minimal improvements in QoL
Chen et al.^25^	China	IG: 213 CG: 210	**IG:** Education-based intervention - Lectures - Periodical follow- up interviews - Annual physical examination - Special medical services **CG:** Routine services	1 year	Not stated	F2F	Lecture: Prevention and self-management strategies, nutrition, PA, health-seeking behaviour and psychological counselling	2 hours every 2 months	Physicians, nurses, public health physicians and DM specialists from county-level hospitals, township health centres and village clinics	EQ-5D-3L EQ-5D-3L dimension EQVAS Baseline 1 year	EQ-5D-3L index: ↑↓ EQ-5D-3L dimension: Mobility: ↑↓ Self-care: ↔ Usual activities: ↑↓ Pain/discomfort: ↔ Anxiety/depression: ↑↓ EQVAS: ↔
Christoffersen et al	Denmark	IG: 234 CG: 76	**IG:** Tailored education using NEED based on participant **CG:** Group-based educational programmes other than NEED, including methods to facilitate interaction, person-centredness and social learning	1 year	Group	F2F	Focus on psychosocial issues and behavioural changes	2 to 4 sessions	Specially trained healthcare professionals	EQ-5D-5L Baseline 3 months 1 year	EQ-5D-5L: ↔
Estuningsih et al.^27^	Surabaya	Grouped according to nutritional education Hospitalised patients IG: 10 CG: 10	Standard medical care and nutritional consultation **IG:** Plus intensive monitoring using a self-regulated learning-based nutritional education **CG:** Plus regular education	4 weeks	Not stated	F2F during inpatient continued at home post-discharge	1st week: Information on DM (definition, diagnosis and treatment) and diet 2nd week: Obstacles encountered during the diet programme and formulation of the diet programme 3rd week: Implementation of the plan and assessment of its effectiveness 4th week: Assessment of the plan’s effectiveness	Weekly	Educator	Subjective: WHOQO L-BREF Objective: Blood glucose cholesterol level, BP and BMI Baseline 1 month	Subjective QoL: ↑↓ Objective QoL: ↑↓ QoL: ↑↓
Forjuoh et al.^28^	United States	IG: 101 CG: 95	**IG:** Chronic Disease Self-Management Program in clinical environments and community-based settings **CG:** Usual clinical DM care with Texas Diabetes Council patient education materials	6 weeks	Classroom-based	F2F	Enhanced decision-making, action planning and effective communication	6 sessions	Master trainers/ lay leaders	HRQOL Baseline 6 months 12 months	HRQOL: ↔
Jahromi et al.^29^	Iran	Elderly women IG: 45 CG: 45	**IG:** DSME - Division into three sub-groups (n=15 each group) and enrolment to ^ei^g^ht^ g^rou^p interaction sessions for each sub-group CG: Not stated	8 weeks	^Group^	F2F	Nutrition, stress management, PA, sleep and rest, safety, glycaemic control and self-care	Weekly	Coordinator	WHOQO L-BREF SF-26 Baseline 2 months 3 months	WHOQO L-BREF SF-26: ↑↓
Jaipakdee et al.^30^	Thailand	IG: 203 CG: 200	**IG:** DSMS with a CAI **CG:** Usual health care - Physical examination - Monitoring of blood glucose - Individual health education - Consultation from a registered nurse and/or other healthcare providers	6 months	Group	F2F	DSMS: (I) Educational section about the disease process through a CAI (video) - Knowledge of and food for DM - PA - Foot care - Medication - Complications and stress management - Self-monitoring of clinical indicators - Goals of DM control (II) Condition management and lifestyle changes - Construction of a problem and its definition - Collaborative goal setting and problem-solving - Contracting for change - Continuing support	3 hours monthly	Trained nurses and healthcare staff	Diabetes Quality of Life Brief Clinical Inventory Baseline 3 months 6 months	Diabetes Quality of Life Brief Clinical Inventory: ↑↓
Mahmoud et al.^31^	Arabia	Patients with T2DM on oral hypoglycaemic and/or insulin therapy 99	Psychoeducational programme based on a variety of interactive educational methods, including counselling, demonstration, group discussion and vignette	4 weeks	Group	F2F	DM overview and its complications, self-care, medications and their side effects, lifestyle modification, clarification of myths and misconceptions and coping skills for living with DM	3 hours	Psychoeducator and nurse	RAND-36 Baseline 5 months	Physical functioning: ↔ Role limitations d/t physical health: ↔ Role limitations d/t emotional problems: ↑↓ Energy/fatigue: ↑↓ Emotional well-being: ↑↓ Social functioning: ↑↓ Pain: ↑↓ General health: ↑↓
Mostalà et al.^32^	Egypt	IG: 30 CG: 30	**IG:** - Three sessions of an educational programme including discussion and feedback using lectures (knowledge) and videos (practical) - A copy of the educational programme for **CG:** Routine oral instructions	8 weeks	Group	F2F	1st session: Definition, causes and types of DM 2nd session: Signs and symptoms and treatment of DM 3rd session: Complications and nursing management of DM	Each session: 1 hour thrice weekly	^Nurse^	WHOQO L-WHO Baseline 2 months	WHOQO L-WHO: ↑↓
Pon et al.^21^	Netherlands	IG: 95 CG: 98	Access to an online care platform (e-Vita) to support self-management Usual care involving 2 to 4 visits per year with a practice nurse and at least 1 annual check-up with a general practitioner **IG:** Plus group-based PRISMA **CG:** Plus PRISMA after 6 months	Baseline	Group	F2F	1st session: Individual stories, T2DM, effects of insulin and medication for blood glucose, blood glucose level monitoring and nutrition 2nd session: Complications and personal risk factors, including nutrition, PA and individual DM action plan	3.5 hours for 2 sessions	Practice nurse and a dietician specialising in DM care	EQ-5D-3L: EQ-5D EQVAS Baseline 6 months 12 months	EQ-5D-3L: ↔ (except EQ VAS on the 6 th month)
Sherifali et al.^22^	Canada	IG: 188 CG: 177	Usual DM education (individual or group) provided by nurses and/or dietitians every 3 to 6 months, along with community resources and an accelerometer **IG:** Plus DM health coaching	1 year	Individual	Phone call	Case management and monitoring, DSME/S, behavioural modification, goal setting and reinforcement and general psychosocial support	Weekly phone calls in the 1st 6 months monthly phone calls in the last 6 months	Registered nurse/ certified DM educator	ADDQoL Baseline 6 months 12 months	ADDQoL: ↑↓
Trento et al.^23^	Italy	IG: 25 CG: 25	**IG:** - Assignment into group care divided into 3 groups of 8 to 9 patients each - Group-based educational sessions followed by individual consultations after each session **CG:** Traditional individual consultations	4 years	Group	F2F	Three phases: a) Introduction b) Main content - role-playing with real-life simulations expressing patients’ opinions and life experiences with DM c) Summary and scheduling of next appointments	1-hour for 7 sessions held every 3 to 4 months and repeated 4 years	Physician	DQOL/Mod Baseline 4 years	DQOL/Mod total: ↔ DQOL/Mod satisfaction: ↑↓ DQOL/Mod impact: ↔ DQOL/Mod complications: ↔
Wong et al.^24^	Hong Kong	298	PEP The PEP consisted of generic components (group-based meetings) and disease-specific knowledge and skill components (lecture-based learning sessions)	6 months	Group	F2F	Generic SE enhancement and lifestyle modification Components: Self-management and behavioural modification, healthy diet, PA, problem-solving, self-monitoring, stress management, psychosocial support and networking and communication Disease-specific knowledge and skills Components: Self-care management, medications lòi DM control and contingency management for hypo- and hyperglycaemia	2 hours for 4 to 5 sessions 2.5 hours for 2 sessions	Well-trained healthcare professionals, mainly social workers and dietitians Experienced nurses	SF-12 and SF-6D Baseline 1 year	**SF-12:** Physical functioning: ↔ Physical role: ↔ Bodily pain: ↑↓ General health: ↔ Vitality: ↔ Social functioning: ↔ Emotional role: ↑↓ Mental health: ↔ Physical composite summary: ↔ Mental composite summary: ↔ SF-6D: ↑↓

Arrows indicate significant (↑↓) or non-significant (↔) differences between the interventions and controls unless otherwise stated. ADDQoL: Audit of Diabetes-Dependent Quality of Life 19; BMI: body mass index; BP: blood pressure; CAI: computer-assisted instruction; CG: control group; DDQoL: Diabetes-Dependent Quality of Life; DM: diabetes mellitus; DQOL/Mod: Diabetes Quality of Life/Mod; DSME/S: Diabetes Self-Management Education and Support; DSMS: Diabetes Self-Management Support; DTTP: Diabetes Treatment and Teaching Programme; EQ-5D-3L: EuroQoL Five-Dimensional Scale; F2F: face-to-face; HRQOL: Health related quality of life; IG: intervention group; NEED: Next Education; PA: physical activity; PEP: Patient Empowerment Programme; PRISMA: Proactive Interdisciplinary Self-Management; RAND-36: RAND 36-Item Health Survey 1.0; SE: selfefficacy; SF-12: Short-Form 12 Health Survey Version 2; SF-36: Short-Form 36 Health Survey; SF-6D: Short-Form Six-Dimensional Scale; WHOQOL-BREF/ WHOQOL-WHO: World Health Organization Quality of Life Scale

## Discussion

Patient education plays a vital role in effectively controlling the various distressing clinical conditions related to T2DM.^11^ This scoping review included 15 studies that described the methods and characteristics of patient education for improving the QoL of patients with T2DM, although QoL was not the primary outcome of the study. The educational method, mode, delivery, content and provider as well as the follow-up interval and participant characteristics differed among the reviewed studies. Therefore, we could not provide a formal recommendation on the optimal educational method.

Cunningham et al.^33^ reported significant QoL improvements with the implementation of Diabetes Self-Management Education (DSME). However, we found that one study conveyed a non-significant trend towards greater improvements in QoL using DSME.^19^ Comparatively, two studies that used DSME/S and Diabetes Self-Management Education Support (DSMS) demonstrated non-significant and significant improvements in QoL, respectively.^20,30^ It is difficult to quantify the effects of DSME or DSME/S on QoL owing to the vast array of the mode, delivery, content and provider of interventions. Nevertheless, a particular method may yield significant improvements in QoL.

The greater improvements in QoL in the study by Azami et al.^19^ were suggested to be attributed to the distribution of booklets, usage of movie clips, group-based educational sessions and supervision via weekly phone calls in the intervention group. In comparison, Boels et al.^20^ showed only a slight improvement in QoL, as they used DSME only. Meanwhile, Jaipakdee et al.^30^ indicated significant improvements in QoL owing to the combination of an educational DM section through the usage of a video and learning approach for disease management and lifestyle change. Hence, DSME and DSMS with supplementary educational resources, including booklets, videos or notes, are effective in improving QoL.

For the other methods of self-management education, non-significant improvements in QoL were reported after CDSMP owing to the implementation of education on decision-making, action planning and effective communication instead of selfmanagement for DM care.^28^ Similarly, the Proactive Interdisciplinary Self-Management (PRISMA) programme encompassed holistic education on blood glucose level monitoring, medication, nutrition and physical activity, which yielded non-significant improvements in QoL.^21^ This result is probably attributed to the implementation of the educational programme during the study baseline only. Additionally, the lack of interest and persuasion to enrol in the PRISMA programme might have contributed to the insufficient improvements in QoL.^21^

Baraz et al.^11^ found that the self-care educational programme using Orem’s Self-Care Model positively impacted the overall average QoL of patients, although the increase was not significant in the psychological domain. Thus, they hypothesised that the absence of education on psychological problems leads to non-significant improvements in QoL.^11^ The psychoeducational programme in the study by Mahmoud et al.^31^ showed significant improvements in the emotional domain but non-significant improvements in the physical domain owing to the absence of education on physical health. The PEP in the study by Wong et al.^24^ also yielded non-significant improvements in QoL in the aspect of physical health owing to the absence of education on this domain. Meanwhile, emotional health-related QoL significantly improved, since selfefficacy and psychosocial support were covered in the educational programme.^24^ Therefore, future studies must emphasise the application of education on psychological distress after the diagnosis of DM in combination with education on physical health.

To fulfil patients’ needs, Christoffersen et al.^26^ tailored their educational programme based on NEED. This intervention consisted of physical, practical, social and psychosocial aspects of living with DM to achieve a balanced life. This group-based patientcentred educational programme yielded nonsignificant improvements in QoL.^26^ The health educational lectures in the study by Chen et al.^25^ also improved QoL. However, the variety of baseline data and participant composition contributed to both significant and nonsignificant improvements in QoL.^25^

Intensive monitoring using a self-regulated learning approach and an interactive group session, including discussion and feedback, significantly and effectively improved QoL.^27,29,32^ Although the individual- and group-based educational programmes equally enhanced QoL, the group-based educational programme was more cost-effective, yielded better treatment satisfaction and provided greater support for lifestyle changes.^34^

Face-to-face training was more effective in delivering patient education than educational technology and text messages. Although Sherifali et al.^22^ applied phone coaching, they conducted weekly and monthly phone calls, which may have aided as a supplementary foundation of motivation and adherence. Moreover, a weekly contact hour was more effective in improving QoL,^22,27,29,31,32^ further strengthening the application of continuous monitoring and supervision during interventions.

Herein, no significant adverse events were observed during the interventions, suggesting that the procedures were well tolerated. However, a few studies reported that the levels of education and income contributed to the dropouts.^19,32^ Therefore, well-matched pairing of sociodemographic data, particularly education, motivation, finance and residence (rural/urban), between intervention and control groups is warranted in future studies to reduce the dropout rate during the study period.

This review has two limitations. First, the outcome measures of QoL in the included studies were heterogeneous, which may limit the standardisation of QoL measurement postintervention. Second, we did not formally evaluate the methodological quality and risk of bias of the included studies, as the general purpose of a scoping review is to provide a more comprehensive overview of available evidence.

## Conclusion

There is a broad array of methods of education for improving the QoL of patients with T2DM. Self-management education with supplementary methods, including the use of booklets, videos or notes and supervision and monitoring, can effectively improve QoL. Future studies must emphasise the application of holistic education covering psychological distress after the diagnosis of DM, diet plan and physical health. Group-based education and face-to-face training can also significantly improve QoL.

## Review protocol registration

The review protocol has been registered within the Open Science Framework (https://osf.io/3gk6v).
